# Extensive study of CCN4, VCAM-1, MMP-3, and GM-CSF as reliable markers for disease activity in rheumatoid arthritis

**DOI:** 10.1016/j.jtumed.2024.08.001

**Published:** 2024-08-13

**Authors:** Ahmed T. Yasin, Eman T. Ali, Falah H. Shari, Ali N. Mohammed

**Affiliations:** a Basrah Hospital for Women and Children, Pharmacy Department, Basrah Health Directorate, Basrah, Iraq; b Department of Clinical Laboratory Sciences, College of Pharmacy, University of Basrah, Basrah, Iraq; c College of Pharmacy, Clinical Laboratory Sciences Department, University of Basrah, Basrah, Iraq; d Rheumatology Department, Alsayab Teaching Hospital, Basrah, Iraq

**Keywords:** البروتين المفرز الناجم عن ونت-1, نشاط المرض المشترك 28 إي إس آر, جزيء التصاق الخلايا الوعائية -1, ماتريكس ميلوبروتيناز-3, التهاب المفصل الروماتويدي, DAS28-ESR, Disease activity, MMP-3, Rheumatoid arthritis, VCAM-1, Wnt-1-induced secreted protein-1

## Abstract

**Background:**

The involvement of Wnt-1-induced secreted protein-1 (WISP1/CCN4) in several inflammatory reaction has recently been proposed. Nevertheless, this protein's involvement in rheumatoid arthritis (RA) remains debated. Associations between poorly diagnosed RA and several classical markers derived from demography and biochemistry have been reported.

**Aim:**

We sought to investigate the reliability and effectiveness of serum concentrations of CCN4, vascular cell adhesion molecule-1 (VCAM-1), matrix melloprotenase-3 (MMP-3), and granulocyte-macrophage colony-stimulating factor (GM-CSF) in monitoring and predicting RA and bone damage, and their correlation with RA disease course.

**Methods:**

The study analyzed 128 patients with RA, comprising 68 newly diagnosed and 60 previously diagnosed patients, as well as 60 controls. Biomarker levels were measured with enzyme linked immuno-sorbent assays. Routine laboratory parameters such as serological, clinical, biochemical, and hematological parameters were additionally measured. Demography, anthropometry, and clinical symptom data were collected through interviews and a questionnaire. The joint disease activity score 28 (DAS28) was used to determine disease activity.

**Results:**

Concentrations of four biomarkers were significantly higher in the RA group than the healthy controls. Elevated biomarker concentrations were also observed in patients with high, rather than moderate or low, DAS28-ESR activity status, except for monocyte count, hematocrit (%), and urea level. Furthermore, CCN4 level positively correlated with VCAM-1, MMP-3, and GM-CSF levels, DA-S28-CRP and DAS28-ESR. The levels of three predictive markers, CCN4, VCAM-1, and MMP-3, were elevated in non-treated patients, whereas GM-CSF level showed no difference. The highest area under the curve was 73.3% for CCN4, with 93.3% sensitivity and 64.7% specificity.

**Conclusion:**

Our data suggest that CCN4 can be reliably used to indicate activity and therapeutic response associated with RA, thus facilitating earlier RA diagnosis.

## الملخص

**أهداف البحث:**

تم مؤخرا اقتراح مشاركة البروتين المفرز الناجم عن ونت-1 (ويسب1/سي سي إن 4) في العديد من التفاعلات مع الالتهاب. ومع ذلك، فإن دوره في التهاب المفاصل الروماتويدي لا يزال موضع جدل. تم الإبلاغ عن الارتباطات بين التهاب المفاصل الروماتويدي الذي تم تشخيصه بشكل سيئ وعدد من العلامات الكلاسيكية المستمدة من الديموغرافيا والكيمياء الحيوية. تهدف هذه الدراسة للبحث في موثوقية وفعالية تركيزات مصل "سي سي إن 4"، وجزيء التصاق الخلايا الوعائية -1، وماتريكس ميلوبروتيناز-3، وعامل تحفيز مستعمرة الخلايا المحببة في المراقبة والتقييم لتوقع التهاب المفاصل الروماتويدي وتلف العظام وارتباطهما بمسار المرض.

**طريقة البحث:**

قامت الدراسة بتحليل 128 مريضا مصابا بالتهاب المفاصل الروماتويدي، بما في ذلك 68 تم تشخيصهم حديثا، و60 تم تشخيصهم مسبقا، و60 كمجموعة مراقبة. تم قياس مستويات العلامات الحيوية باستخدام مقايسة الممتص المناعي المرتبط بالإنزيم، وتم قياس المعلمات المخبرية الروتينية مثل المعلمات المصلية والسريرية والكيميائية الحيوية والدموية. تم جمع بيانات الديموغرافيا والقياسات البشرية والأعراض السريرية من خلال المقابلات والاستبيان، وتم استخدام درجة نشاط المرض المشترك 28 لتحديد نشاط المرض.

**النتائج:**

أشارت النتائج إلى ارتفاع معنوي في تراكيز أربعة مؤشرات حيوية في مجموعة التهاب المفاصل الروماتويدي مقارنة بالعينة السليمة. كما تم تسجيل تراكيز مرتفعة في المرضى الذين يعانون من ارتفاع، مقارنة مع المرضى المعتدلين والمنخفضين، وفقا لحالة نشاط "المرض المشترك 28 إي إس آر"، باستثناء عدد الوحيدات، ونسبة الهيماتوكريت، ومستوى اليوريا. علاوة على ذلك، تم تسجيل ارتباط إيجابي بين مستوى "سي سي إن 4" وقيم جزيء التصاق الخلايا الوعائية -1، ماتريكس ميلوبروتيناز-3، عامل تحفيز مستعمرة الخلايا المحببة، درجة نشاط "المرض المشترك 28 سي آر بي" ودرجة نشاط "المرض المشترك 28 إي إس آر". مستويات العلامات التنبؤية الثلاثة ("سي سي إن 4" وجزيء التصاق الخلايا الوعائية -1 وماتريكس ميلوبروتيناز-3) كانت مرتفعة في المرضى غير المعالجين، في حين أن مستوى عامل تحفيز مستعمرة الخلايا المحببة لم يظهر أي فرق.

**الاستنتاجات:**

تشير هذه البيانات إلى أنه يمكن استخدام "سي سي إن 4" بشكل موثوق لتحديد النشاط والاستجابة العلاجية المتعلقة بالتهاب المفاصل الروماتويدي، مما يؤدي إلى تشخيص التهاب المفاصل الروماتويدي مبكرا.

## Introduction

In contrast to many other autoimmune conditions, rheumatoid arthritis (RA) is chronic and systemic. In terms of pathology, RA is characterized by continuous inflammatory synovitis, pannus development, and excessive lymphocyte infiltration. Subsequently, the cartilage and bone tissues in the joint are destroyed. RA develops under the influence of several risk factors, such as infectious, hormonal, and hereditary factors.^1^ Response to therapy is routinely monitored through specific indicators, primarily the disease activity score in 28 joints (DAS28) and C-reactive protein (CRP).^2^ Responses to therapy vary, thus strongly indicating the involvement of various biochemical pathways in RA development.^3^^,^^4^ Hence, the current work focused on investigating key biomarkers to improve prediction of the therapeutic response.

Optimization of treatment and the determination of long-term prognosis require several steps, first and most importantly determining RA prognosis as early as possible. RA can be distinguished from various other entities affecting the joints according to serological marker testing. RA can be treated if early diagnosis is achieved, and the disease enters remission in approximately 50% of cases.^5^ Having infections often leads to false positive test results, which can also occur in response to alterations in thyroglobulin, alpha-fetoprotein, sex hormone binding proteins, troponin 1, various cytokines, hepatitis B virus serology, and latex agglutination. In the case of autoimmune reactions, test findings can be affected by the concentrations of autoantibodies in the plasma. Rheumatoid factor (RF) is detected in approximately 60–80% of patients with chronic RA, but only approximately 50–60% of those with an earlier RA diagnosis. RA ultimately occurs in only 11–20% of patients with musculoskeletal complaints and positive RF test results.^6^ Although both ACPAs and RFs have fairly accepted values for diagnosis, the discovery of other markers is necessary to improve RA diagnosis.^7^ Among those markers, CCN4 has unfortunately not been included in relevant recently published literature reviews.^8^^,^^9^ Nevertheless, after the introduction of multiple-biomarker disease activity (MBDA) testing for RA in 2016, the number of subjective indicators have used has increased. The MBDA is composed of 12 serum biomarkers (vascular cell adhesion molecule-1 (VCAM-1), epidermal growth factor (EGF), vascular endothelial growth factor A (VEGF-A), Interleukin-6 (IL-6), tumor necrosis factor receptor (TNFRI), matrix melloprotenase-1 (MMP-1), matrix melloprotenase-3 (MMP-3), leptin, resistin, and CRP). The MBDA has recently been demonstrated to mirror RA activity, and to provide a prediction of radiological progression and the risk of flares after drug reduce. In testing of RA serological markers, several markers have been subjected to extensive investigations of their potential to reflect disease activity, monitor progress, and track patient responsiveness to therapy. However, these markers have received insufficient of interest among most specialists in this field.^10^

Recent studies have focused on finding evidence for understanding the contribution of the CCN family to RA. Increasing knowledge of the mechanisms through which this family of proteins pathologically and physiologically influences the course of several forms of arthritis might result in the development of therapeutic strategies that successfully target CCN functions and molecular pathways, and alleviate morbidity in patients. Wnt-1-induced secreted protein-1 (WISP1/CCN4) is a member of the CCN family of matricellular proteins, which also includes cysteine-rich 61 (Cyr61/CCN1), connective tissue growth factor (CTGF/CCN2), nephroblastoma overexpressed gene (NOV/CCN3), Wnt-1-induced secreted protein-2 (WISP-2/CCN5), and Wnt-1-induced secreted protein-3 (WISP-3/CCN6).^11^ VCAM-1 has high significant in prediction of the risk of osteoarthritis (OA) development.^12^

Although this molecule influences inflammation-associated events during the development of RA, few studies have described the relationship between alterations in VCAM-1 levels and disease activity in RA.^13^

RA cannot be controlled in all cases by blocking individual cytokines or cell types. Therefore, alternative cytokines or mechanisms contributing to RA pathogenesis must urgently be studied.^14^ Granulocyte-macrophage colony-stimulating factor (GM-CSF) is a growth factor known to regulate hemopoiesis^15^ and to be crucial in regulating the activity of mature myeloid cells, including macrophages. Preclinical investigations, as well as observations of arthritis flares after treatment with GM-CSF, have supported the crucial role of this molecule in pathogenesis. Most clinicians use GM-CSF for the treatment of post-chemotherapy neutropenia. Researchers have raised concerns that treatment protocols targeting GM-CSF might have severe side effects, including neutropenia and pulmonary alveolar proteinosis. This molecule plays crucial roles in processes through which macrophages differentiate, survive, and are activated. Therefore, inhibition of its activity might affect macrophage function and possibly confer clinical advantages in patients with RA.^14^ Moreover, MMP3 is an enzyme studied for its ability to reflect disease activity, monitor disease progress, and track therapeutic responsiveness. However, the level of interest in this enzyme to date has been insufficient. Most MMPs are secreted as inactive pre-proteins, whose activation is achieved via cleavage by extracellular proteinases. Among these, MMP3 has proteinase functions, and is synthesized and secreted by synovial fibroblasts and chondrocytes. This enzyme has an active role in the destruction of joints in RA.^16^

The present work is novel because serum CCN4 levels in patients with RA can be used to monitor the efficiency of treatment in responsive, newly diagnosed patients during early disease stages, before therapies that could cause irreversible damage are administered. Herein, we aimed at assessing serum levels of CCN4, VCAM-1, MMP-3, and GM-CSF in patients with RA; their relationship with disease activity; and the most important risk factors. Hence, the current work focused on investigating key biomarkers to better predict the response to therapy, and on evaluating the reliability and effectiveness of these molecules as biomarkers for monitoring and prediction of RA activity, and joint and bone damage.

## Materials and Methods

### Study design

**Figure 1:**
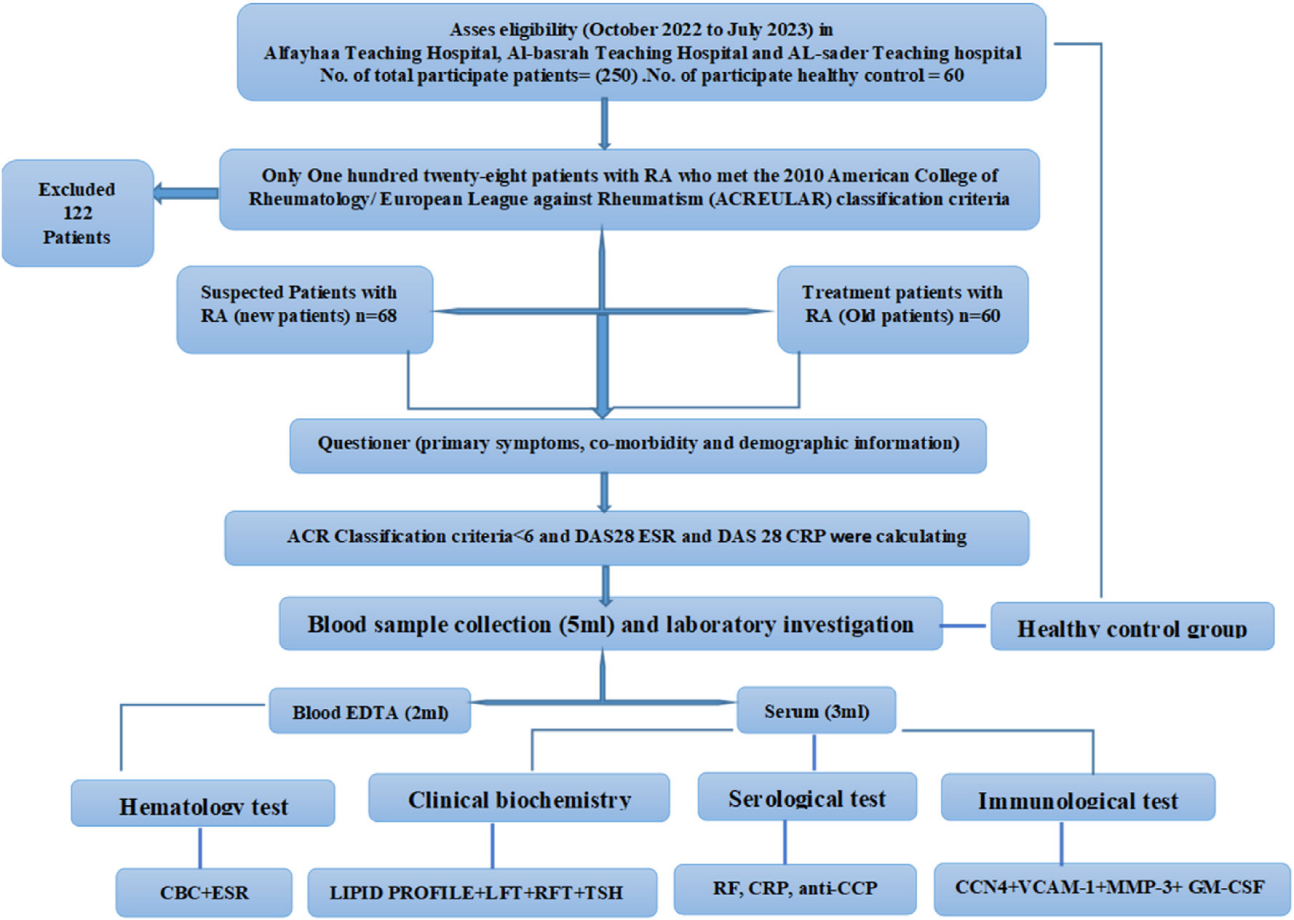
Flowchart of study design.

The present work constituted a cross-sectional investigation from October 2022 to July 2023. The study included 250 adult men and women (20–73 years of age). Only 128 patients with RA meeting the 2010 American College of Rheumatology/European League against Rheumatism (ACR/EULAR) classification criteria^17^ were chosen from rheumatology consulting clinics. Clinical RA diagnosis was conducted by a specialist rheumatologist visiting the Al-Fayhaa teaching hospital, Al-Basrah teaching hospital, and Al-Sadder teaching hospital in Basrah, Iraq (see Figure 1).

### Patients and control participants

Of the 128 patients with RA, 68 were newly diagnosed and suspected of having had RA for more than 6 months (and had not started any clinical and serological investigations); these patients ranged in age from 27 to 73 years. RA was clinically confirmed by a specialist rheumatologist, and these patients were classified as group I (new patients with RA). On the basis of patient status, nonsteroidal anti-inflammatory drugs (piroxicam capsules, 20 mg; naproxen tablets, 500 mg; celecoxib capsules, 200 mg; diclofenac tablets, 50 mg; and meloxicam tablets, 15 mg) were prescribed as treatments. Sixty patients with a previous diagnosis of RA (serologically positive), ranging in age from 20 to 70 years, were diagnosed according to ACR/EULAR classification criteria^17^ with disease with more than 12 months’ duration; these patients were classified as group II (pre-existing patients). These patients were selected during a routine visit to the Basra General Hospital's Biological Treatment Center and received various treatment protocols, including disease modifying anti-rheumatic drugs (DMARDs), non-steroidal anti-inflammatory drugs (NSAIDs), steroids, and biological treatments. The total number of patients (128) was divided into three subgroups (low, moderate, or high) according to disease severity (DAS28-ESR). These patients were compared with the healthy group comprising 60 healthy men and women, matched in age range (24–71 years), who were recruited among the hospital staff and healthy volunteer relatives from various areas. The exclusion criteria were identical to those of the patients. Diagnosis, treatment, and follow-up were performed in the abovementioned hospitals. Data collection was achieved via interviews based on a questionnaire created by the researchers. In each clinic, a single investigator evaluated all patients' clinical histories and physical examination findings. The list of parameters analyzed in the preliminary tests included demographic data, clinical findings, disease activity (number of sensitive or swollen joints among 28 joints), drug history, presence of articular and extra-articular symptoms, presence of concomitant comorbid diseases, and outcomes of laboratory examinations.

### Retrieval of demographic and anthropometric data

The demographic and anthropometric data included age, body weight, height, family history, menopausal status, disease duration, clinical symptoms, and marital status. Physical examination information included joint pain, joint swelling, morning stiffness, and spindle shape. Body mass index was calculated with the World Health Organization (WHO) approach, as the patient's height in meters squared divided by the weight in kilograms. The WHO classification system was used to categorize patients into underweight (less than 18.5 kg/m^2^), normal (18.5–25 kg/m^2^), overweight (25 to <30 kg/m^2^), or obese (>30 kg/m^2^) BMI.^18^

### Exclusion criteria

The exclusion criteria were advanced cases with severe joint deformity; age below 20 years; pregnancy; other conditions of autoimmunity; acute severe infection; acute cardiovascular disease; diabetes mellitus; hypertension; malignancy; smoking; treatment with angiotensin-converting enzyme inhibitors, oral contraceptives, or statins; and diseases potentially causing dyslipidemia, e.g., hypothyroidism and nephrotic syndrome.

### Routine laboratory assessment

#### Blood sample collection

A 5 mL volume of whole blood was withdrawn by venipuncture from patients with RA during their morning visit to the Consulting Clinic/Al-Fayhaa General Hospital (before starting the treatment protocol) or when they attended the Biological Therapy Center/Basra General Hospital for biological therapy. Similarly, 5 mL blood samples were withdrawn from the control group. A 2 mL volume of the blood sample was placed in an ethylene diaminetetraacetic acid tube (AFCO, Jotden) and directly used for complete blood count analysis, and 3 mL was placed in plain gel tubes (AFCO, Jotden). The whole blood samples were allowed to clot at room temperature (18–25 °C) for 15–30 min. Subsequently, serum was separated by centrifugation at 1,000–2,000×*g* for 10 min. Serum aliquots (1.5 mL) were kept in micro-centrifuge tubes and subsequently divided into three Eppendorf tubes (Eppendorf, Germany) containing 0.5 mL each, then stored in a −80 °C freezer (Vest frose, Denmark) at the Consulting Clinic/Al-Fayhaa General Hospital until analysis of the studied markers.

### Hematology profiles

The vital hematological parameters were measured in 2 mL blood in a test tube coated with ethylene diaminetetracetic acid anticoagulant. An automated hematology analyzer (Ruby, Germany) was used to perform the complete blood count and measuring the absolute counts of white blood cells (WBC), neutrophils, monocytes, lymphocytes, and red blood cells (RBC), along with hemoglobin level. Hemoglobin levels in patients with anemia should be 12.0 g/dl for adult women and 13.0 g/dl for adult men, according to WHO reference values.^19^

### Erythrocyte sedimentation rate tests

As a non-specific screening examination, the erythrocyte sedimentation rate (ESR) was calculated by using the Westergren test. Data are expressed in units of millimeters per hour (mm/hr).^20^

### Clinical biochemistry tests

Sera from venous blood specimens (approximately 2 mL in plain tubes) were used to assess kidney and liver function; determine the concentrations of serum creatinine, uric acid, and urea; and perform, liver function tests (alanine transaminase (ALT), total bilirubin, direct bilirubin, aspartate aminotransferase (AST), and alkaline phosphatase (ALKP)) with an Abbott diagnostic (Architect, USA) fully automated system, according to the manufacturer's instructions.

## Estimation of CRP, RF, cyclic citrullinated peptides, and thyroid-stimulating hormone

Measurement of CRP, RF, anti-cyclic citrullinated peptides (anti-CCP), and thyroid-stimulating hormone (TSH) in the sera of patients and controls was performed with a Cobas E 411 analyzer (Roche, Germen) as a fully automated system, according to the manufacturer's instructions (test CRP4X, test ID 0-736; test RF-II, test ID 0-757; Elecsys Anti-CCP, test number 810; TSH test number 1820). The normal reference values were 0–5 mg/dL for CRP; 0–14 IU/mL for RF; 0–20 IU/mL for anti-CCP; and. 0.27–4.2 μIU/mL for TSH.

### Enzyme linked immunosorbent assays

Evaluation of the serum concentrations of human CCN4, VACM-1, and MMP3 proteins, and the cytokine GM-CSF, was performed with enzyme linked immunosorbent assays (ELISAs) with commercially available kits, according to the manufacturer's protocol (Elabscience, USA).

### Assessment of RA activity

DAS28 was determined according to the evaluation of swollen joint count in 28 joints (SJC28), whereas the tender joint count in 28 joints is referred to as TJC28. The global assessment used the general visual analog scale, with a scoring system from 0 (best) to 100 (worst). Finally, the calculation of DAS28-ESR was achieved with the following formulas:

DAS28-ESR = 0.56√ (TJC28) + 0.28 √ (SJC28) + 0.70 Ln (ESR) + 0.014 (GH).

DAS28 (CRP) = 0.56∗√ (TJC28) + 0.28∗√ (SJC28) + 0.014∗ GH + 0.36 ∗ln (CRP+1) + 0.9 6 RA disease activity was determined according to the following scores: remission (DAS28 ≤ 2.6), low activity LDA (2.6 < DAS28 ≤ 3.2), moderate activity MDA (3.2 < DAS28 ≤ 5.1), and high activity HAD (DAS28 > 5.1).

### Statistical analysis

For statistical analysis of the collected data, SPSS version 26 (SPSS Inc., Chicago, IL, USA) was used. Qualitative data are reported as numbers and percentages. Testing of quantitative data for normality of distribution was performed with the Shapiro–Wilk and Kolmogorov–Smirnov tests. Parametric data are reported as mean ± SD (SD), whereas non-parametric data are reported as median with minimum and maximum values, in addition to mean ± SD. Variations between two groups of parametric data were tested for significance with Student's t-test, whereas significance between non-parametric data groups was investigated with the Mann–Whitney U test. For more than two groups of non-parametric data, the Kruskal–Wallis test was applied. To investigate correlations between quantitative data sets, we used the Spearman non-parametric correlation test. Equations for significant correlations are shown in simple linear regression curves. Sensitivity and specificity were measured for the targeted markers, and the ROC curve and area under the curve (AUC) are presented.

Positive predictive value (PPV) = (TP/(TP + FP) × 100) and negative productive value (NPV) = (TN/(TN + FN) × 100) were determined by application of multivariate binary logistic regression analysis for the assessment of certain risk factors’ effects on the erosion and development of RA disease. P-values less than 0.05 were considered significant.

## Results

Table 1 shows the distribution of patients according to sociodemographic, anthropometric, and medical information, categorized according to whether the patient was new or pre-existing. Among 128 patients, 106 (82.8%) were women, and 22 (17.2%) were men. The average age, disease duration, and body mass index were 48.9 ± 11.5 years, 43.3 ± 12.0 months, and 30.2 ± 4.5 kg/m^2^, respectively. The age distribution showed insignificant variations between the new and pre-existing RA patient groups (p = 0.100), with a higher proportion of participants 40–59 years of age. Insignificant differences in BMI values were also observed. Patients in the new and pre-existing groups who were obese accounted for the highest proportion (50.0%, 46.6%, respectively); P = 0.030. Regarding residential address, the highest proportions in both groups were from urban areas, with comparable percentages (70.1%, 29.7%, respectively) to rural areas (p = 0.002). Menopause status differed significantly (p = 0.010) between the new and pre-existing RA patient groups. Most patients (56.3%) were postmenopausal, whereas 28.1% were premenopausal (p = 0.002). Moreover, 68 (53.1%) of patients with RA had a family history involving a first-degree relative with the disease. The values of RF and anti-CCP seropositivity rates were 77.3% and 79.6%, respectively (p ≤ 0.050), and 76.8% of all patients with RA were seropositive RF+ anti-CCP.

**Table 1:** Demographic and disease features of patients with rheumatoid arthritis.

Characteristics	New patients with RA	Pre-existing patients with RA	Total patients with RA	P-value
	N = 68	N = 60	N = 128	
Demographic	∗N (%)	N (%)	N (%)	
Age (year)				
∗∗Mean ± SD	50.0 ± 12.01	47.7 ± 10.88	48.9 ± 11.5	0.100
Minimum–maximum	(27–73)	(20–70)	(20–73)	
20–39	12 (17.74)	8 (13.4)	20 (15.6)	0.020
40–59	42 (61.76)	46 (76.6)	88 (68.8)	
≥60	14 (20.5)	6 (10)	20 (15.6)	
Sex				
Male	12 (17.6%)	10 (16.7)	22 (17.2)	0.001
Female	56 (82.4%)	50 (83.3)	106 (82.8)	
Address				
Rural	21 (30.9)	18 (30.0)	38 (29.7)	0.002
Urban	47 (69.1)	42 (70.0)	90 (70.3)	
Duration of disease				
Mean ± SD	2.0 ± 0.4	84.9 ± 21.5	43.3 ± 12.0	0.002
Range, month	(0.5–12)	(24–240)	(0.5–240)	
Family history of RA in a first-degree relative				
Positive	36 (52.9)	32 (53.3)	68 (53.1)	0.010
Negative	32 (47.1)	28 (46.7)	60 (46.9)	
Menopausal state				
Premenopausal	18 (26.5)	17 (28.3)	36 (28.1)	0.002
Post menopause	38 (55.9)	34 (56.7)	72 (56.3)	
∗∗BMI	30.2 ± 3.6	30.1 ± 5.4	30.2 ± 4.5	0.200
Normal	4 (5.8)	4 (6.6)	8 (6.25)	0.03
Underweight	0	2 (3.34)	2 (1.5)	
Overweight	30 (44.1)	26 (43.3)	56 (43.7)	
Obese	34 (50)	28 (46.66)	62 (48.4)	
RF positivity	43 (63.2)	56 (93)	99 (77.3)	0.002
Anti-CCP positivity	60 (88.2)	42 (70)	102 (79.6)	0.003
Seropositivity (RF + anti-CCP)	41 (60.2)	42 (70)	83 (76.8)	0.001

∗Frequency and percentages (n, %) are used to display categorical variables. ∗∗Continuous data are shown as mean ± SD.

As shown in Table 2, significant differences were found in almost all factors and markers tested in all patients with RA and controls, except for HCT, platelets, serum TSH, blood urea, and serum HDL, whose levels did not significantly differ between these groups. The indicators that were higher in patients with RA were BMI, total WBC count, neutrophil count, lymphocyte count, monocyte count, RBC count, ALT level, AST level, alkaline phosphatase level, direct bilirubin level, total bilirubin level, triglyceride level, total serum cholesterol level, LDL level, random blood glucose level, anti-CCP level, CRP level, RF level, ESR level, CCN4 level, VCAM1 level, MMP3 level, and GM-CSF level. The indicators that were significantly higher in controls included MCH level, MCV level, HGB level, and serum creatinine level, as shown in Table 2 (p ≤ 0.050).

**Table 2:** Comparison of demographic, hematological, clinical biochemical, inflammatory, and immunological parameters among the studied groups.

Parameter	Total patients with RA	Healthy controls	P-value
	N = 128	N = 60	
Sex, female/male	106/22	33/27	0.0001^a^
%	(82.8%)/(17.2%)	(55%)/(45%)	
Age mean ± SD (year)	48.9 ± 11.5	49.6 ± 12.0	0.100
Range	(20–73)	(24–71)	
BMI (kg/m^2)^	30.2 ± 4.5	26.1 ± 7.1	0.0001
WBC (10^3^/μL)	7.4 ± 2.6	6.4 ± 1.8	0.008
Neutrophils (10^3^/μL)	4.5 ± 3.8	3.1 ± 1.3	0.0001
Lymphocytes (10^3^/μL)	2.3 ± 0.8	1.9 ± 0.9	0.003
Monocytes (10^3^/μL)	0.6 ± 0.4	0.5 ± 0.2	0.034
RBCs (10^6^/μL)	4.6 ± 0.4	4.7 ± 1.0	0.100
HGB (g/dL)	12.1 ± 1.4	13.7 ± 1.0	0.010
HCT (%)	38.7 ± 4.0	39.1 ± 4.5	0.500
MCV (fL)	81.2 ± 14.6	89.8 ± 2.9	0.010
MCH (pg)	26.1 ± 3.1	28.7 ± 0.9	0.100
PLT (10^3^/μL)	297.0 ± 104.3	277.6 ± 64.4	0.300
TSH (μIU/mL)	2.8 ± 0.9	3.0 ± 1.9	0.100
S-Creatinine (mg/dL)	0.6 ± 0.1	0.7 ± 0.1	0.100
Urea (mg/dL)	24.8 ± 8.1	24.3 ± 7.8	0.900
ALT (U/L)	23.1 ± 11.2	16.8 ± 8.0	0.010
AST (U/L)	20.8 ± 7.3	17.5 ± 4.5	0.002
D.BILI (mg/dL)	0.31 ± 0.1	0.2 ± 0.10	0.010
T.BILI (mg/dL)	0.6 ± 0.3	0.4 ± 0.1	0.0001
ALKP (U/L)	97.8 ± 37.6	73.0 ± 49.4	0.001
T. cholesterol (mg/dL)	176.0 ± 43.7	138.2 ± 30.7	0.001
LDL (mg/dL)	108.8 ± 31.7	86.9 ± 15.7	0.0001
HDL (mg/dL)	48.1 ± 13.5	46.1 ± 9.4	0.100
Triglyceride (mg/dL)	147.6 ± 66.5	84.8 ± 33.1	0.0001
Glucose (mg/dL)	118.2 ± 40.9	102.4 ± 26.2	0.0100
Anti-CCP (IU/mL)	27.6 ± 11.6	4.0 ± 1.5	0.0001
CRP (mg/dL)	17.8 ± 7.7	3.1 ± 0.8	0.0001
RF (IU/mL)	20.0 ± 10.5	7.5 ± 1.8	0.0001
ESR (mm/hr)	38.44 ± 26.	14.3 ± 5.1	0.0001
CCN4 (pg/mL)	4437.7 ± 1726.6	439.5 ± 205.8	0.0001
VCAM-1 (ng/mL)	1743.2 ± 1192.4	302.5 ± 65.4	0.0001
MMP3 (ng/mL)	256.0 ± 154.6	52.5 ± 22.2	0.0001
GM-CSF (pg/mL)	126.5 ± 52.7	3.0 ± 1.9	0.0001

Data are mean ± SD. ∗P value is significant at 0.050.

BMI: Body Mass Index; WBC: White Blood Cells; RBC: Red Blood Cells; HGB: Hemoglobin; HCT: Hematocrit; MCV: Mean Corpuscular Volume; MCH: Mean Corpuscular Hemoglobin; PLT: Platelets; TSH: Thyroid Stimulating Hormone; ALT: Alanine Transaminase; AST: Aspartate Aminotransferase; ALKP: Alkaline Phosphatase; BILID: Direct Bilirubin; BILIT: Total Bilirubin; LDL: Low Density Lipoprotein; HDL: High Density Lipoprotein; Anti-CCP: Anti-Cyclic Citrullinated Peptide; CRP: C-Reactive Protein; RF: Rheumatoid Factor; ESR: Erythrocyte Sedimentation Rate; DAS28-ESR: Disease Activity Score 28-ESR; DAS28-CRP: Disease Activity Score28-CRP; CCN4: Wnt-1 Induced Secreted Protein-1 (WISP1/CCN4); VCAM-1: Vascular Cell Adhesion Molecule 1; MMP-3: Matrix Metalloproteinase-3; GM-CSF: Granulocyte-macrophage Colony-Stimulating Factor.

When the activity of rheumatoid arthritis was considered, levels of all studied factors were statistically significantly different according to DAS28-ESR activity status, except for monocyte count, HCT (%), and urea level (Table 3). The mean values of DAS28-ESR and DAS28-CRP significantly differed (P = 0.0001) (Table 3). The average value of DAS28-ESR in patients with RA with HAD was 5.8 ± 0.5, and was higher than that in patients with LDA and MDA (2.73 ± 0.07, 4.4 ± 0.5, respectively); in contrast, the mean value of DAS28-CRP was 4.8 ± 0.5 in patients with HAD (P = 0.0001) (Table 3). We observed higher serum levels of CCN4, VCAM-1, MMP3, and GM-CSF proteins in patients with RA with HAD (6133.3 ± 763.3 pg/mL, 2994.1 ± 788.2 ng/mL, 356.9 ± 175.4n g/mL, and 180.2 ± 31.5 pg/mL, respectively) rather than with LDA and MDA (P = 0.0001). The results also revealed a significantly lower average concentrations of hemoglobin in the patients (11.86 ± 1.25 g/dL) than the healthy participants (P = 0.001). This finding applied to the values of the inflammatory factors as well as the serum concentrations of anti-CCP, CRP, RF, and ESR in patients with RA with HAD (28.9 ± 10.5 IU/mL, 19.305 ± 12.6 mg/dl, 20.1 ± 10.1 IU/mL and 52.12 ± 18.09 mm/h, respectively), which were higher than those in patients with LDA and MDA (P = 0.0001), as indicated in Table 3.

**Table 3:** Comparative analysis of serum levels of CCN4, VCAM-1, MMP3, and GM-CSF, and inflammatory, hematological, and clinical biochemistry parameters in patients with RA, divided into groups classified according to DAS28-ESR, compared with healthy controls.

Parameter	Total patients with RA, N = 128			Healthy controls	P-value
	Low	Moderate	High		
	N = 4	N = 74	N = 50	N = 60	
Sex, F/M	2/2	54/20	50/0	33/27	∗0.0001
%	(1.5)/3.6	40.6/36.4	37.6%	55%/45%	
Age, year	62.5 ± 10.9	48.57 ± 12.6	48.4 ± 9.0	49.6 ± 12.0	∗0.0001
Range	(53–72)	(20–73)	(28–69)	(24–71)	
BMI (kg/m^2^)	28.9 ± 2.6	29.6 ± 4.4	31.2 ± 4.7	26.1 ± 7.1	∗0.0001
WBCs (10^3^/μL)	6.5 ± 0.6	7.5 ± 2.1	7.22 ± 1.77	6.47 ± 1.80	0.0300
Neutrophils (10^3^/μL)	3.6 ± 0.9	4.8 ± 4.7	4.1 ± 1.8	3.1 ± 1.3	∗0.001
Lymphocytes (10^3^/μL)	2.5 ± 0.0	2.3 ± 0.7	2.4 ± 0.8	1.9 ± 0.9	∗0.010
Monocytes (10^3^/μL)	0.4 ± 0.2	0.7 ± 0.4	0.6 ± 0.1	0.5 ± 0.2	∗0.030
RBCs (10^6^/μL)	5.3 ± 0.1	4.6 ± 0.5	4.5 ± 0.3	4.7 ± 1.07	∗0.0001
HGB (g/dL)	14.2 ± 0.4	12.1 ± 1.4	11.8 ± 1.2	13.7 ± 1.0	∗0.0001
HCT (%)	46.3 ± 2.3	38.6 ± 3.9	38.1 ± 3.6	39.1 ± 4.5	∗0.010
MCV (fL)	86.7 ± 2.5	81.8 ± 11.5	79.8 ± 18.8	89.8 ± 2.9	∗0.0001
MCH (pg)	26.7 ± 0.2	26.1 ± 2.8	26.1 ± 3.7	28.7 ± 0.9	∗0.0001
PLT (10^3^/μL)	197.5 ± 30.6	283.3 ± 118.7	325.2 ± 72.4	277.65 ± 64.4	∗0.0001
TSH (μIU/mL)	1.7 ± 0.86	2.7 ± 1.0	3.0 ± 0.9	2.5 ± 0.8	∗0.008
S. Creatinine (mg/dL)	0.6 ± 0.0	0.6 ± 0.1	0.59 ± 0.1	0.7 ± 0.1	∗0.001
Urea (mg/dL)	19.7 ± 4.9	25.9 ± 9.1	23.7 ± 6.4	24.3 ± 7.8	0.500
ALT (U/L)	27.5 ± 4.0	25.8 ± 5.4	0.3 ± 0.1	0.2 ± 0.1	∗0.020
AST (U/L)	17.5 ± 6.3	21.26 ± 7.29	20.57 ± 7.66	17.55 ± 4.55	∗0.009
D.BILI (mg/dL)	0.2 ± 0.0	0.3 ± 0.1	0.3 ± 0.1	0.2 ± 0.1	∗0.0001
T.BILI (mg/dL)	0.3 ± 0.05	0.6 ± 0.3	0.6 ± 0.3	0.4 ± 0.1	∗0.001
ALKP (U/L)	157.5 ± 106.8	90.2 ± 33.3	104.2 ± 30.0	73.0 ± 29.4	∗0.0001
T. Cholesterol (mg/dL)	155.0 ± 11.5	166.0 ± 46.5	192.5 ± 35.4	138.2 ± 30.7	∗0.0001
LDL (mg/dL)	89.5 ± 1.7	103.57 ± 30.34	118.10 ± 32.86	86.92 ± 15.72	∗0.0001
HDL (mg/dL)	41.5 ± 0.5	47.1 ± 12.5	50.1 ± 15.2	46.1 ± 9.4	∗0.020
Triglycerides (mg/dL)	103.5 ± 0.5	136.4 ± 52.6	157.8 ± 74.7	84.8 ± 33.1	∗0.0001
Glucose (mg/dL)	99.7 ± 4.1	112.4 ± 47.8	132.1 ± 28.6	102.4 ± 26.2	∗0.010
Anti-CCP (IU/mL)	19.6 ± 9.4	27.2 ± 12.3	28.9 ± 10.5	4.0 ± 1.5	∗0.0001
CRP (mg/dL)	2.0 ± 1.9	12.43 ± 20.81	19.305 ± 12.67	3.11 ± 0.82	∗0.0001
RF (IU/mL)	10.0 ± 2.2	20.4 ± 10.7	20.1 ± 10.1	7.5 ± 1.8	∗0.0001
ESR (mm/hr)	5.0 ± 0.7	31.0 ± 11.9	52.1 ± 18.0	14.30 ± 5.1	∗0.0001
DAS28-ESR	2.7 ± 0.07	4.4 ± 0.5	5.8 ± 0.5	–	∗0.0001
DAS28-CRP	2.9 ± 0.1	3.94 ± 0.6	4.86 ± 0.5	–	∗0.0001
CCN4 (pg/mL)	834.5 ± 30.1	3486.8 ± 1094.7	6133.3 ± 763.3	439.5 ± 205.8	∗0.0001
VCAM-1 (ng/mL)	583.0 ± 19.4	960.7 ± 139.0	2994.1 ± 788.2	302.5 ± 65.4	∗0.0001
MMP-3 (ng/mL)	87.4 ± 0.3	196.9 ± 94.1	356.9 ± 175.4	52.5 ± 22.2	∗0.0001
GM-CSF (pg/mL)	66.3 ± 1.3	93.5 ± 29.9	180.2 ± 31.5	3.02 ± 1.9	∗0.0001

Data are mean ± SD. ∗P value is significant at 0.05.

Significant differences in serum levels on the basis of the DAS28-ESR and DAS28-CRP were observed (P = 0.001). Table 4 provides a summary of the outcomes of comparisons of DAS28-ESR and DAS28-CRP. At baseline, a higher number of patients met the higher disease activity criteria (>5.1) for DAS28-ESR than DAS28-CRP. The levels in patients with HAD were higher than those in patients with other disease activity degrees (remission, LDA, and MDA), as shown in Table 4.

**Table 4:** Associations of serum concentrations of CCN4, VCAM-1, MMP-3, and GM-CSF with disease activity in the group of new patients with RA.

Disease activity		CCN4	VCAM-1	MMP-3	GM-CSF
DAS28-ESR	LDA	∗834.5 ± 30.13	583.0 ± 19.4	87.4 ± 0.34	66.3 ± 1.38
	MDA	2993.1 ± 1020.9	1034.9 ± 630.2	181.9 ± 69.8	108.5 ± 28.0
	HAD	5757.3 ± 452.9	2627.4 ± 534.5	368.5 ± 46.41	182.2 ± 22.1
	P-value	0.0001∗∗	0.0001∗∗	0.0001∗∗	0.0001∗∗
DAS28-CRP	Remission	565.7 ± 111.3	693.7 ± 121.1	237.4 ± 99.1	83.0 ± 32.2
	LDA	1458.4 ± 581.6	621.0 ± 36.30	108.2 ± 19.43	77.5 ± 13.6
	MDA	4081.8 ± 1451.2	1628.8 ± 907.1	249.0 ± 100.5	137.1, P ± 39.1
	HAD	5316.8 ± 1546.5	2426.8 ± 1212.8	348.6 ± 139.0	172.5 ± 56.9
	P-value	0.0001∗∗	0.0001∗∗	0.0001∗∗	0.0001∗∗

∗Results are presented as mean ± SD. ∗∗P value is considered significant at or below 0.050.

LDA: Low disease activity; MDA: Moderate disease activity; HAD: High disease activity.

Remission (DAS28 ≤ 2.6), LDA (2.6 < DAS28 ≤ 3.2), MDA (3.2 < DAS28 ≤ 5.1), and HAD (DAS28 > 5.1).

Serum levels of CCN4, VCAM-1, and MMP-3 showed significant differences according to treatment modality (P = 0.001, P = 0.050, and P = 0.010, respectively). All predictive markers (CCN4, VCAM-1, and MMP-3) showed elevated levels in non-treated patients, except for GM-CSF. These data confirmed that the lowest concentrations of CCN4 and MMP-3 were observed in patients treated with biological protocols, whereas the lowest level of VCAM-1 was observed in patients treated with DMARD protocols (Table 5).

**Table 5:** Association of serum levels of CCN4, VCAM-1, MMP-3, and GM-CSF with treatment protocol and patient response in the group of treated patients with RA.

Treatment protocol	CCN4	VCAM1	MMP3	GM-CSF
Negative	∗6223.5 ± 2164.1	2239.8 ± 1437.4	434.4 ± 87.4	120.11 ± 55.2
NSAIDs	5603.1 ± 1607.2	2184.9 ± 1269.5	427.1 ± 359.1	121.17 ± 45.0
DMARDs	4268.8 ± 869.9	1172.4 ± 736.9	239.6 ± 142.2	134.73 ± 55.2
Steroid	5179.0 ± 1437.2	1839.1 ± 1457.3	326.43 ± 232.6	110.7 ± 39.26
Biological	3577.3 ± 1480.1	1377.7 ± 909.5	219.8 ± 138.3	112.7 ± 75.71
DMRD + steroid	4999.1 ± 420.1	1299.5 ± 1094.1	227.2 ± 99.4	144.83 ± 64.3
NSAID + DMARD	5199.1 ± 1346.7	1679.5 ± 867.4	231.6 ± 71.03	112.5 ± 22.51
∗P-value	0.0001∗∗	0.05∗	0.01∗	0.580

Data are mean ± SD.∗P value is significant at 0.050.

NSAID: Non-steroidal anti-inflammatory drug; DMARD: Disease-modifying antirheumatic drug.

As shown in Table 6, in patients with RA, the ESR and DAS28-CRP levels significantly correlated with CCN4, VCAM1, MMP3, GM-CSF, and DAS28-ESR levels. A significant correlation was observed between CRP and DAS28-ESR values (r = 0.245, P = 0.040). All these correlations were directly proportional (Table 6).

**Table 6:** Assessment of correlations among serum levels of CCN4, VCAM-1, MMP-3, GM-CSF, and DAS28-ESR with inflammatory markers and DAS28-CRP.

Parameter	PC	CCN4	VCAM-1	MMP3	GM-CSF	DAS28-ESR
RF (IU/mL)	r	0.379	0.179	0.100	0.170	0.143
	P	0.01	0.144	0.416	0.165	0.244
CRP (mg/dL)	r	0.224	0.120	0.056	0.236	0.245∗
	P	0.066	0.331	0.648	0.052	0.044
Anti-CCP (IU/mL)	r	0.202	0.135	0.128	0.174	0.183
	P	0.023	0.273	0.297	0.156	0.136
ESR (mm/hr)	r	0.485∗∗	0.451∗∗	0.342∗∗	0.458∗∗	0.459∗∗
	P	0.0001	0.0001	0.004	0.0001	0.0001
DAS28-CRP	r	0.779∗∗	0.763∗∗	0.753∗∗	0.817∗∗	0.851∗∗
	P	0.0001	0.0001	0.0001	0.0001	0.0001

∗Correlation is considered significant at or below a probability value of 0.05 (two-tailed).

∗∗Correlation is considered significant at or below a probability value of 0.01 (two-tailed).

Attempts to find correlations among CCN4, VCAM1, GM-CSF, MMP3, and DAS28-ESR levels with TSH, LDL, ALKP, age, and BMI values in patients with RA indicated generally significant statistical correlations, except for CCN4 with ALKP, and MMP3 with ALKP. All correlations were directly proportional, except for correlations with age, which were inversely proportional (Table 7).

**Table 7:** Assessment of correlations of serum levels of CCN4, VCAM-1, MMP3, and GM-CSF with various parameters.

Parameter	PC	TSH	LDL	ALKP	Age	BMI
DAS28-ESR	r	0.538∗∗	0.349∗∗	0.232	0.289∗	0.381∗∗
	P	0.0001	0.004	0.05	0.01	0.001
CCN4 (pg/mL)	r	0.573∗∗	0.435∗∗	0.179	0.278∗	0.257∗
	P	0.0001	0.0001	0.144	0.02	0.035
VCAM-1 (ng/mL)	r	0.516∗∗	0.390∗∗	0.289∗	0.243∗	0.416∗∗
	P	0.0001	0.001	0.01	0.045	0.0001
MMP3 (ng/mL)	r	0.583∗∗	0.357∗∗	0.159	0.293∗	0.321∗∗
	P	0.0001	0.003	0.195	0.015	0.008
GM-CSF (pg/mL)	r	0.519∗∗	0.331∗∗	0.247∗	0.255∗	0.351∗∗
	P	0.0001	0.006	0.04	0.03	0.003

∗Correlation is considered significant at or below a probability value of 0.05 (two-tailed).

∗∗Correlation is considered significant at or below a probability value of 0.01 (two-tailed).

**Figure 2:**
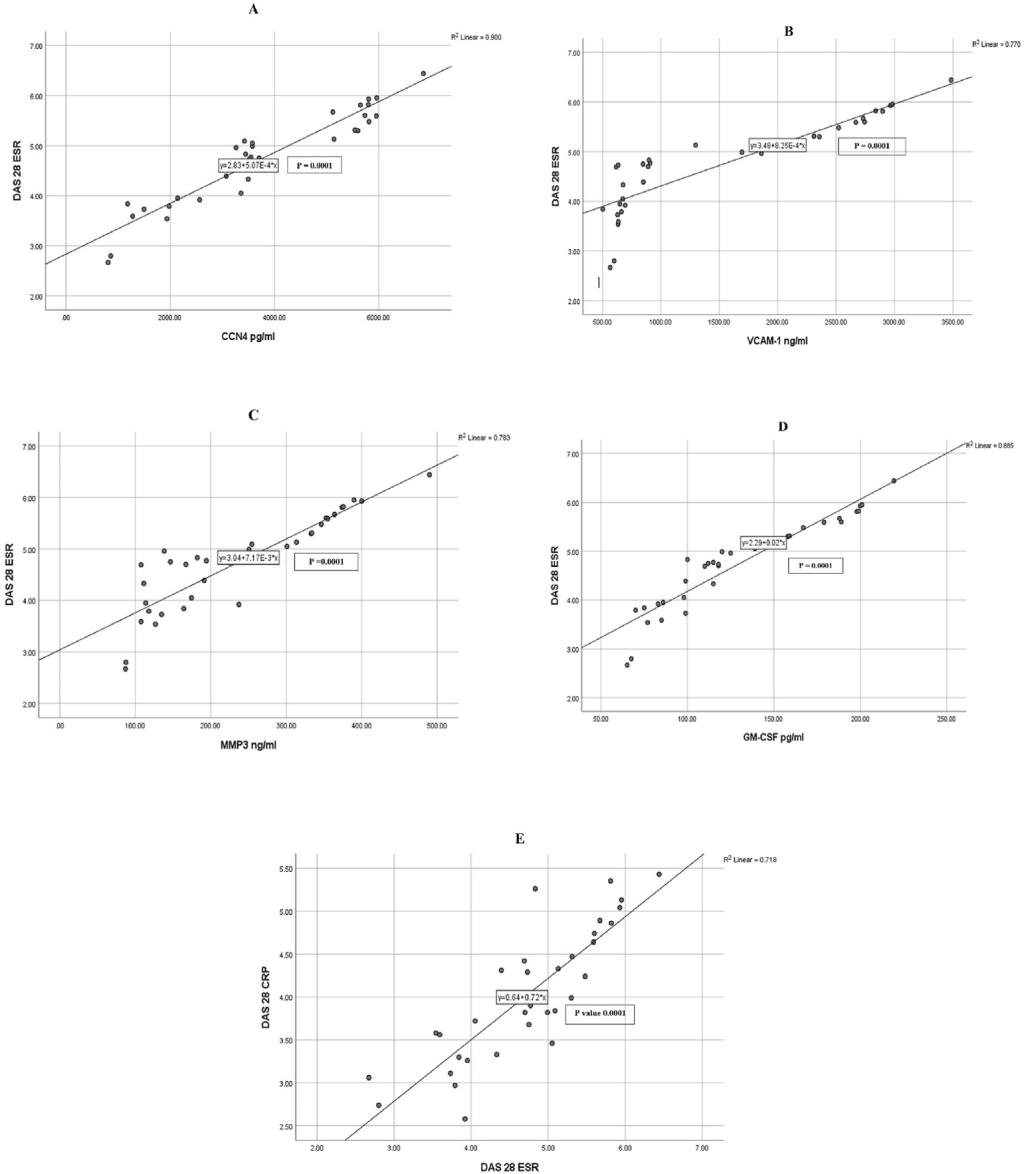
Assessment of regression between DAS28-ESR and predictable inflammatory markers. A: CCN4, B: VCAM-1, C: MMP3, D: GM-CSF, and E: DAS28-CRP.

**Figure 3:**
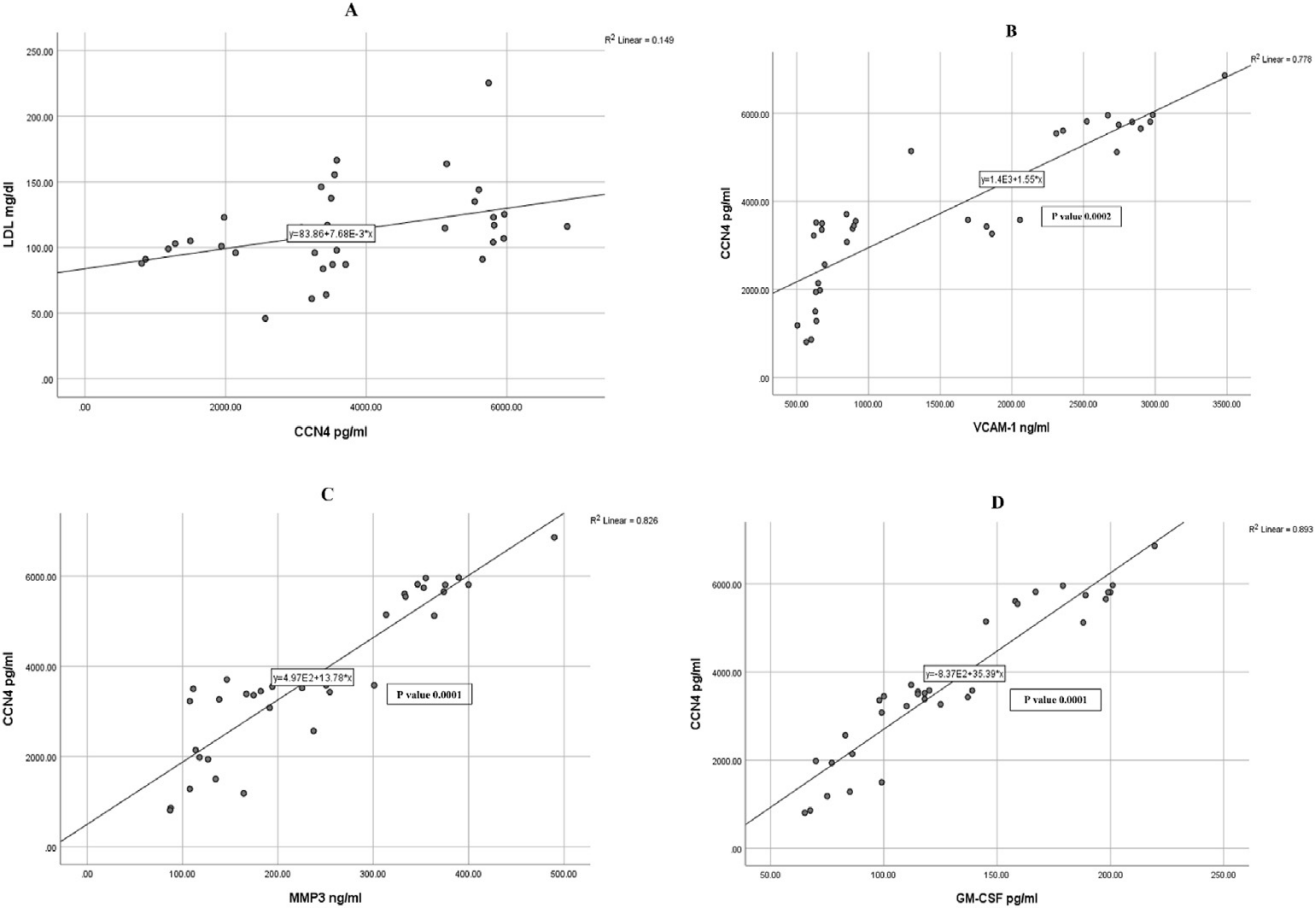
Assessment of regression between CCN4 and predictable inflammatory markers. A: LDL, B: VCAM-1, C: MMP-3, D: GM-CSF.

Figure 2, Figure 3 show a graphical presentation of significant correlations among the studied quantitative variables. In addition, they show simple linear regression equations between two correlated variables.

**Figure 4:**
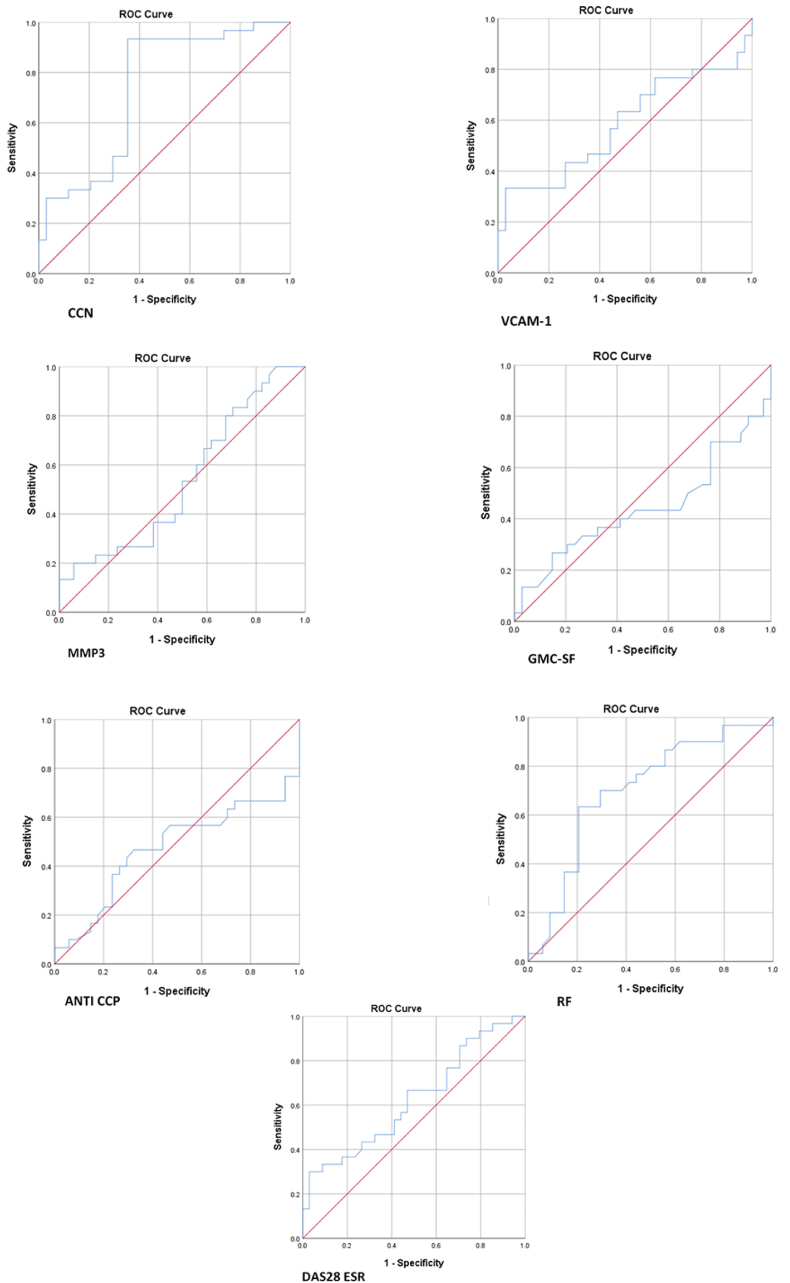
Receiver operating characteristic analysis of the predictive biomarkers. A: CCN4, B: VCAM-1, C: MMP-3, D: GM-CSF, E: Anti-CCP, F: RF, G: DAS28-ESR.

To explore the possibility of using the indicators as screening markers for RA, we determined the AUC for each indicator. As shown in Figure 4, receiver operating characteristic curve analysis of the indicators CCN4, VCAM-1, MMP3, GM-CSF, anti CCP, RF, and DAS28-ESR indicated AUC values of 73.7%, 58.3%, 53.8%, 44.2%, 47.4%, 70%, and 62.3%, respectively. The AUCs were statistically significant with a 95% CI, as shown in Table 8.

**Table 8:** Receiver operating characteristic analysis of the predictive biomarkers.

Predictive biomarker	AUC	Std. error^a^	Sig.^b^	95% confidence interval	
				Lower bound	Upper bound
CCN4	∗∗0.737	0.045	0.0001∗∗	0.649	0.826
VCAM-1	0.583	0.053	0.104	0.480	0.686
MMP3	0.538	0.052	0.456	0.437	0.639
GM-CSF	0.442	0.053	0.256	0.338	0.546
Anti-CCP	0.474	0.054	0.606	0.368	0.579
RF	∗∗0.700	0.047	0.0001∗∗	0.607	0.793
DAS28-ESR	∗∗0.623	0.050	0.017∗	0.526	0.721

AUC: Area Under the Curve; Std. Error: Standard Error; Sig: Significant.

a Under the nonparametric assumption.

b Null hypothesis: true area = 0.5.

Table 9 clarifies that the cut-off value of serum CCN4 at 3828.9 pg/mL had 93.3% sensitivity and 64.7% specificity. Its predictive measures were 70% PPV and 91.7% NPV. The AUC of RF was 70%, thus confirming the reliability 70% (60.7%–79.3%). This area was statistically significant, and the results were reliable. The serum RF cut-off value of 18.75 IU/mL had a sensitivity of 63.3% and a specificity of 79.4%. The predictive measures were 73.1% PPV and 71.1% NPV. Finally, regarding validity, DAS28-ESR showed a sensitivity of 30% and specificity of 97.1%; regarding predictivity, it showed a 90% PPV and 61.6% NPV. These results led to a 65.7% total agreement rate and a 34.4% total misclassification rate (Table 9).

**Table 9:** Sensitivity, specificity, positive predictive value, negative predictive value, total agreement rate, and total misclassification rate of predictive biomarkers in screening RA.

Predictive biomarker	∗Optimal cutoff value	Sen. (%)	Spec. (%)	PPV	NPV	TAR	TMR
CCN4	3828.9	a93.3	64.7	70.0	a91.7	a78.2	21.9
VCAM-1	3003	33.3	a97.1	a90.9	c62.3	67.2	32.9
MMP3	390.6	20.0	c94.1	75.0	57.1	59.4	40.6
GM-CSF	189.5	26.5	85.3	61.5	56.9	57.8	42.2
Anti-CCP	31.5	46.7	67.6	56.0	59.0	57.8	42.2
RF	18.75	b63.3	79.4	73.1	b71.1	b71.9	28.1
DAS28-ESR	5.3	30.0	b97.1	b90	61.1	65.7	34.4

P-value is significant at 0.05. ∗Positive if greater than or equal to optimal cutoff value.

Sen: Sensitivity; Spec: Specificity; PPV: Positive Predictive Value; NPV: Negative Predictive Value; TAR: Total Agreement Rate; TMR: Total Misclassification Rate.

a high percentage

b moderate percentage

c low percentage

## Discussion

The present work involved a comprehensive study exploring which biomarkers are reliable in terms of disease activity monitoring, predictive ability, and indication of therapeutic response in RA. Some typical clinical, demographic characteristics and serological data of Iraqi RA patients were also investigated. Our patient Iraqi RA sample showed the same demographic, clinical, and serological data distribution patterns as those in studies from other areas worldwide,^21^^,^^22^ and had high similarity to those from developing countries and Brazil.^23^ Our results revealed higher disease prevalence in women, a finding attributed to the influence of sex hormones and their complicated interactions with immune responses.^24^

Because no research has assessed the role of CCN4 in RA, a better understanding of the mechanisms through which CCN4 proteins influence the pathophysiological mechanisms associated with various types of arthritis is needed. According to our findings in Table 2, overall, patients with RA had significantly higher levels of CCN4, VCAM-1, MMP-3, and GM-CSF proteins than healthy participants (p = 0.0001). The outcome data indicated elevated CCN4 levels in patients with RA with HDA. We further observed a direct association with RA activity, as indicated by DAS28, which showed higher values in patients with elevated RA activity than in patients with moderate or low disease activity. Furthermore, CCN4 levels showed positive associations with VCAM-1, MMP-3, GM-CSF, DAS28-CRP, and DAS28-ESR. Furthermore, a positive correlation was observed between CCN4 and the levels of several clinical biochemistry parameters, such as TSH and LDL, as well as demographic characteristics, e.g., age and BMI. Moreover, our study indicated greater mean serum CCN4 levels in non-treated patients with RA than patients with RA treated with various protocols. More precisely, the study indicated a decrease in the mean serum CCN4 level (3577.31480.1 pg/mL) after biological therapy initiation with respect to baseline levels (6223.52164.1 pg/mL); P = 0.050. VCAM-1 expression is strongly associated with RA.^25^ The present data indicated a significantly higher VCAM-1 levels in patients than healthy participants, in agreement with previously reported findings.^26^ Serum VCAM-1 levels increased, as did serum MMP-3, GM-CSF, anti-CCP, RF, ESR, and CRP. The VCAM-1 level in the blood reflected the altered state of RA and, to a certain degree, response to therapy. The current data support that RA, a chronic inflammatory disease, is associated with elevated levels of VCAM-1, a protein that significantly increases the secretion of proinflammatory cytokines.^27^ In this context, Pulito et al. have discussed how the induction of VCAM-1 is caused by proinflammatory cytokines and hypoxia.^28^ VCAM-1 affects immune system function, and is involved in lymphocyte formation and immune system adjustment. Elevated VCAM-1 in the serum has been associated with immune system disorders.^29^ Additionally, elevated serum VCAM-1 levels are found in patients with high, rather than moderate or low, RA activity. A correlation between lower VCAM-1 levels in treated than new patients with RA, on the one hand, and RA activity and responsiveness to treatments, on the other hand, is proposed. This study confirmed that the lowest level of VCAM-1 was observed in patients treated with a DMARD protocol than in patients in the other groups. More precisely, lower VCAM-1 levels (1172.4 ± 736.9 ng/mL) were found after DMARD therapy initiation than were observed in non-treated patients with RA (2239.8 ± 1437.4 ng/mL; P < 0.050). This finding was in agreement with those from previous studies.^30^^,^^31^ The immune system gradually recovers normal function as the symptoms and inflammatory reactions lessen, thus causing the serum VCAM-1 level to gradually decrease.^27^

According to our findings, DAS28-ESR and serum VCAM-1 in patients with RA were significantly positively correlated, in agreement with findings from a prior study.^32^ A noteworthy finding indicated that VCAM-1 and CCN4, MMP-3, GM-CSF, DAS28-CRP, ESR, TSH, and LDL levels; age; and BMI were significantly positively correlated. This result is somewhat consistent with those from a recent study,^32^demonstrating elevated VCAM-1 in patients with RA, in correlation with other parameters such as disease activity, oxidative stress, and inflammatory markers.

The role of CCN4 in RA has not been fully revealed. CCN4 uses autocrine mechanisms for accelerating cell growth, inducing transformation in morphology, elevating saturation density, and stimulating tumorigenesis.^33^ In addition, CCN4 stimulates osteoblasts to differentiate.^34^ Furthermore, the critical functions of CCN4 during several cellular processes, such as cell proliferation, adhesion, migration, and differentiation, along with the regulation of extracellular matrix differentiation, are well documented.^34^ According to a previous investigation by Liu et al., the action of CCN4 in increasing VCAM-1 secretion is mediated through its interaction with the αvβ5/α6β1 integrin receptor and the subsequent activation of Syk, PKCδ, and JNK, thereby enhancing AP-1 binding, trans-activation of VCAM-1 expression, and monocyte adhesion to OASFs.^35^ Despite the yet uncertain RA mechanisms of pathogenesis, extensive investigations have revealed that the migration of mononuclear cells makes an essential contribution to the preservation of synovial inflammation.^36^ Adhesion molecules, such as VCAM-1, regulate the processes through which these cells adhere to, and infiltrate into, sites of inflammation.^37^ These molecules comprise transmembrane glycoproteins that mediate interactions among cells, as well as between cells and their extracellular matrix. VCAM-1 exerts adhesion functions via its involvement in multiple vital activities associated with normal physiology, as well as disease pathology. These activities include WBC and vascular cell viscosity during inflammation, immune cell recognition, lymph node homing, tumor invasion and metastasis, and intracellular signal transduction.^38^ We presumed that CCN4 was responsible for the elevated serum VCAM-1 levels. Because synovial samples are difficult to obtain, and are typically unreliable in the clinical diagnosis of RA, the present work instead measured serum levels of predictive biomarkers. In addition, those biomarkers exert local production in inflamed joints and subsequently are secreted into the circulation. An association has been proposed between biomarker levels in the serum and in the synovial fluid, thereby reflecting the degree of activity of rheumatoid synovitis.^39^

The effects of GM-CSF on RA patient outcomes is currently unknown and requires further study. Except in cases of RA, GM-CSF levels are elevated in joints; thus, inhibiting GM-CSF as a biological target might decrease inflammatory or damaging reactions.^40^ Our data indicated that serum GM-CSF levels are elevated in RA patients with HDA in comparison with patients with moderate or low disease activity (in terms of DAS28), compared with control. Furthermore, GM-CSF levels were positively correlated with VCAM-1, MMP-3, DAS28-CRP, and DAS28-ESR. In addition, GM-CSF levels correlated with demographic data such as age and BMI, as well as clinical biochemical parameters such as TSH, LDL, and ALKP. A previous study in patients with RA^14^ has reported expression of GM-CSF in the synovial membrane, and elevated GM-CSF levels in synovial fluid.^41^ GM-CSF plays a crucial role in the processes through which macrophages differentiate, survive, and are activated. Therefore, the inhibition of GM-CSF activity might influence macrophage function and confer clinical advantages in RA. GM-CSF is not a growth factor with roles solely in the proliferation of myeloid cells; it is also crucial in the regulation of the functions of mature myeloid cells, including chemotaxis and cell adhesion, dendritic cell function, expression of cytosis, and microbial killing.^42^^,^^43^

Finally, all patients with RA showed higher MMP-3 serum levels than the controls (p = 0.0001). This finding is largely similar to previously reported findings.^44^^,^^45^ Correlation analysis between MMP-3 and age revealed a significant correlation (p = 0.010), in contrast to findings from a prior study.^45^ In addition, we found higher MMP-3 levels in the total sample of patients with HAD than in patients with moderate or low disease activity as well as controls. A significant correlation was found between these levels and disease activity, expressed in terms of DAS28. This finding is consistent with those from a prior study^46^ and reflects the degree of synovitis.^47^ Because the investigations targeted the relationship between MMP-3 and RA treatment, the present work aimed at examining this connection to predict treatment responsiveness in RA. In this context, our results revealed that serum MMP-3 levels were significantly correlated exclusively with ESR (p = 0.001). This finding supports those from a prior study^46^ reporting a significant correlation between MMP-3 and several laboratory parameters, namely ESR, CRP, and RF. However, the correlation between MMP-3 and anti-CCP was insignificant. Peake et al. have reported that MMP-3 levels had an insignificant correlation with ESR, as supported by the present work.^48^ More precisely, the mean MMP-3 levels decreased after the onset of the biological treatment (219.8 ± 138.3 ng/mL), in comparison to the baseline value (434.4 ± 87.44 ng/mL; P < 0.050), in agreement with prior findings^4^ indicating that this elevation at the time of diagnosis is attributable to the effects of activated inflammatory cytokines that induce synovial cells and neutrophils to enhance MMP-3 expression.^49^ These findings support that elevated MMP-3 might reflect disruptive mechanisms within the joints, thus indicating poor prognosis during earlier stages of RA.^50^ Outcome data in RA are closely associated with disease activity.^51^ Therefore, disease activity scores are routinely used to provide guidance regarding individual treatment protocols and to assess the efficiency of treatment in clinical trials. The present study revealed significant differences in CCN4, VCAM-1, MMP-3, and GM-CSF levels according to disease severity (as indicated by both DAS28-ESR and DAS28-CRP). In parallel, the bulk of research in the past decade has indicated a decreased DAS28-CRP value in comparison with that of DAS28-ESR.^52^^,^^53^

To explore the possibility of using the indicators for screening; reliable predictive markers of disease activity; or follow-up markers for RA, we determined the AUC for each indicator. Higher AUC indicates better model discrimination between tests, as shown in the graphs. The total number of patients tested was 128, of whom 60 were treated patients with RA, and 68 were newly diagnosed untreated patients with RA. CCN4 had the highest AUC value, followed by RF and DAS28-ESR. The remaining values were considered negligible, including anti-CCP and GM-CSF. CCN4 and other biomarkers were tested with receiver operating characteristic curve analysis, and yielded an AUC value of 73.7%. Consequently, when CCN4 sensitivity and the false positive rate were linked, they confirmed reliability 73.7%. Furthermore, the significant p-value suggested a significant relationship between the model and outcome, in agreement with previous studies^54^,^55^. Furthermore, in a study by Hattori et al., at the 113 ng/mL value of sMMP-3, the sensitivity and specificity were 57.32% and 78.57%, respectively, in discriminating patients who achieved remission.^40^ Hattori et al. have reported a limit value of MMP-3 of less than 76.7 ng/mL, with a sensitivity of 47.27%, a specificity of 83.05%, and an AUC of 63.73% after remission^56^. Our data showed differences with respect to those from other studies in terms of the cut-off, sensitivity, and specificity values of biomarkers used to discriminate between RA patient groups. This variability was caused by several factors, including the sample size, age, types of kits used in biomarker tests, treatment types, and follow-up periods. According to our results, CCN4 is a protein that might stimulate the production of other predictive markers, and that contributes primarily to the inflammatory process of RA, and leads to joint erosion and destruction. Therefore, it may be used as a confirmation test by rheumatologists to follow up on patient condition and to use instead of RF for diagnosis.

On the basis of these findings, we hypothesize that CCN4 plays a crucial role in the development of RA and could be used as a potential biomarker of RA activity. This biomarker might result in future therapeutic approaches that focus on the functions and mechanisms of action of this molecule, and may decrease patient morbidity. Notably, the present findings can be considered reliable; all study participants were age and sex matched, and no comorbidities occurred. This comprehensive study highlights the importance of all inflammatory, serological, and biochemical indicators in diagnosing RA, in contrast to previous studies that focused on specific parameters.

The limitations of this study were its small sample size; lack of assessment of other CCN family members; and reliance on serum instead of knee synovial fluid. Further studies should be performed to examine intracellular signaling pathways and genetic variation in the *WISP1* gene in a large number of patients with RA.

## Conclusions

In conclusion, our study suggests that CCN4, VCAM-1, MMP-3, and GM-CSF may be useful predictive biomarkers for identifying patients with RA. In particular, CCN4 may serve as a valid biomarker for disease activity, disease outcome predictability, and therapeutic response in RA, thus contributing to the early diagnosis of RA.

## Source of funding

This research did not receive any specific grant from funding agencies in the public, commercial, or not-for-profit sectors.

## Conflict of interest

The authors declare no conflicts of interest.

## Ethical approval

Ethics approval was obtained from the Research Committee/Training and Human Development Center of the Basra Health Department, Ministry of Health, according to resolution No. 270/2022 dated October 26, 2022. All participants provided written informed consent.

## Authors contributions

ATY: Conceptualization, Methodology, Software. ANM: Diagnosis. ATY and ETA: Data curation, Writing—Original draft preparation. ETA: Project administration. ATY: Visualization, Investigation. ETA and FHS: Supervision.: ATY: Software, Validation: ATY and ETA: Writing—Reviewing and Editing. All authors have critically reviewed and approved the final draft and are responsible for the content and similarity index of the manuscript.
